# Autoantibodies Produced at the Site of Tissue Damage Provide Evidence of Humoral Autoimmunity in Inclusion Body Myositis

**DOI:** 10.1371/journal.pone.0046709

**Published:** 2012-10-05

**Authors:** Arundhati Ray, Anthony A. Amato, Elizabeth M. Bradshaw, Kevin J. Felice, Daniel B. DiCapua, Jonathan M. Goldstein, Ingrid E. Lundberg, Richard J. Nowak, Hidde L. Ploegh, Eric Spooner, Qian Wu, Simon N. Willis, Kevin C. O’Connor

**Affiliations:** 1 Department of Neurology, Human and Translational Immunology Program, Yale School of Medicine, New Haven, Connecticut, United States of America; 2 Department of Neurology, Harvard Medical School and Brigham and Women’s Hospital, Boston, Massachusetts, United States of America; 3 Center for Neurologic Diseases, Brigham and Women’s Hospital, Harvard Medical School, Boston, Massachusetts, United States of America; 4 Neuromuscular Program, Hospital for Special Care, New Britain, Connecticut, United States of America; 5 Department of Neurology, University of Connecticut School of Medicine, Farmington, Connecticut, United States of America; 6 Department of Neurology, Division of Neuromuscular Medicine, Yale School of Medicine, New Haven, Connecticut, United States of America; 7 Rheumatology Unit, Department of Medicine, Karolinska University Hospital in Solna, Karolinska Institutet, Stockholm, Sweden; 8 Whitehead Institute for Biomedical Research, Department of Biology, Massachusetts Institute of Technology, Cambridge, Massachusetts, United States of America; 9 Department of Pathology and Laboratory Medicine, University of Connecticut Health Center, Farmington, Connecticut, United States of America; Children's Hospital Boston/Harvard Medical School, United States of America

## Abstract

Inclusion body myositis (IBM) belongs to a group of muscle diseases known as the inflammatory myopathies. The presence of antibody-secreting plasma cells in IBM muscle implicates the humoral immune response in this disease. However, whether the humoral immune response actively contributes to IBM pathology has not been established. We sought to investigate whether the humoral immune response in IBM both in the periphery and at the site of tissue damage was directed towards self-antigens. Peripheral autoantibodies present in IBM serum but not control serum recognized self-antigens in both muscle tissue and human-derived cell lines. To study the humoral immune response at the site of tissue damage in IBM patients, we isolated single plasma cells directly from IBM-derived muscle tissue sections and from these cells, reconstructed a series of recombinant immunoglobulins (rIgG). These rIgG, each representing a single muscle-associated plasma cell, were examined for reactivity to self-antigens. Both, flow cytometry and immunoblotting revealed that these rIgG recognized antigens expressed by cell lines and in muscle tissue homogenates. Using a mass spectrometry-based approach, Desmin, a major intermediate filament protein, expressed abundantly in muscle tissue, was identified as the target of one IBM muscle-derived rIgG. Collectively, these data support the view that IBM includes a humoral immune response in both the periphery and at the site of tissue damage that is directed towards self-antigens.

## Introduction

Inclusion body myositis (IBM) is an idiopathic inflammatory myopathy. It is a progressive skeletal muscle disorder and has a distinct clinical phenotype that includes weakness and atrophy of distal and proximal muscles [1]–[4]. The clinical course progresses slowly and often leads to severe debilitation. The etiology and pathogenesis of IBM remain poorly understood, but aging, genetics and environment may each play a role. The salient features of disease pathology are inflammation and myodegeneration [5]. IBM muscle biopsies are characterized by an infiltration of CD8+ T cells associated with MHC class I expression by the muscle fibers, vacuolization of muscle fibers and accumulation of protein aggregates (termed inclusion bodies) in the cytoplasm and nucleus [6]. Nodular collections of cells are found within the endomysial and perimysial space [7]. It remains unclear whether IBM is primarily a T cell-mediated or myodegenerative disease [6], [8], but each is thought to contribute to the disease pathology [9].

The other major inflammatory myopathies, polymyositis and dermatomyositis, include humoral autoimmunity evidenced by the presence of autoantibodies and response to B cell depleting therapy [10], although experience with the latter is limited. In IBM, the humoral immune response has been studied to a lesser extent than the T cell response because B cells and antibody-secreting plasma cells were not, until recently, known to be present in the damaged tissue. However, an abundance of differentiated B cells, in the form of CD138^+^ plasma cells, that populate the injured tissue were recently identified [11], [12]. The molecular characterization of these muscle-associated plasma cells indicated that these cells were antigen-experienced and clonally expanded [7], [12]. Furthermore, serum autoantibodies, in a subset of patients with IBM, have been reported to react with a number of self-antigens [13], [14] with one study showing that approximately half of IBM patients appear to harbor serum autoantibodies that react with an unknown muscle antigen [15]. Finally, BAFF, a molecule crucial for B cell maturation, survival and autoantibody production, is elevated in a subset of patients with IBM [16]. These data suggest that IBM may include humoral autoimmunity in addition to the established T cell component of immunopathology.

Here we sought to examine whether the humoral immune response in the periphery and at the site of muscle tissue damage in IBM patients is directed towards self-antigens. We examined serum-derived immunoglobulin for reactivity to both tissue and tissue-derived cell lines. Immunohistochemistry revealed that IBM serum IgG but not control IgG bound antigens in muscle tissue, indicating that IBM serum harbors an immune response directed to self-antigens. This was further confirmed with flow cytometry on human cell lines. In addition, we examined the reactivity of the local antibody response in IBM muscle tissue by generating antibodies derived from individual plasma cells that were isolated from the damaged tissue. Here we established that IBM muscle-derived recombinant IgG (rIgG), but not control rIgG, bound antigens expressed by human cell lines and present in muscle tissue homogenates. Furthermore, we identified the intermediate filament Desmin, a reported autoantigen in a number of autoimmune conditions [17]–[19], as a target of the immunoglobulin produced within the IBM muscle tissue. Collectively, these data establish that humoral autoimmunity directed towards self-antigens is present both in the periphery and at the site of tissue damage in IBM patients.

## Materials and Methods

### Ethics Statement

Specimens originating from patients were collected after informed written consent was obtained, under a protocol approved by the Human Research Protection Program at Yale School of Medicine. Specimens, that did not include personally identifiable private information or intervention or interaction with an individual, were collected under an exempt protocol approved by the Human Research Protection Program at Yale School of Medicine.

### Patient-derived Specimens

Serum-derived IgG was obtained from 9 patients with IBM and 9 normal subjects. Collected sera were stored at −80°C. IgG was purified from serum using Melon Gel IgG Purification kits (Thermo Scientific) according to the manufacturer’s instructions. This approach to isolation of IgG from serum was favored over protein-G/A because Melon Gel does not require the use of harsh elution conditions that can contribute to denaturation of the immunoglobulins. IBM muscle biopsies, collected from four patients, were snap frozen and stored at −80°C immediately after collection. Biopsies were performed for clinical indications, independent of the current study.

### Cell Lines and Culture Conditions

Muscle-derived cell lines CRL-1598 and CCL-136, obtained from ATCC, were cultured at 37°C, 5% CO_2_ in a humidified chamber. Cell lines were grown in high glucose DMEM media (Gibco) supplemented with 4 mM L-glutamine, 1 mM sodium pyruvate and 10% FBS (Gibco). The human oligodendrocyte (HOG) cell line [20] was grown in high glucose DMEM media supplemented with 10% FBS. The media formulation for the HOG cell line was also supplemented with a mixture of penicillin (50 U/mL) and streptomycin (50 µg/mL).

### Flow Cytometry

Cell lines were detached from tissue culture plates by incubation with 10 mM EDTA in PBS for 5 min at 37°C, then washed in PBS. For cell-surface staining, 2×10^5^ cells were resuspended in 50 µl of PBS containing 1% BSA, then incubated with immunoglobulin for 1 hour on ice. Serum-derived IgG was added to each sample to achieve a final concentration of 50 µg/ml, while recombinant IgG were used at 5 µg/ml. The cells were then washed in ice-cold PBS three times, then incubated with goat anti-human IgG conjugated to AlexaFluor 647 (1∶1000; Molecular Probes) for one hour on ice, washed as indicated above then resuspended in PBS. Propidium iodide (PI) (eBioscience) was then added to exclude nonviable cells from the analysis. Cells were analyzed by flow cytometry within 1 hour of addition of PI. Samples for each cytometry were prepared and analyzed on the same day to exclude the introduction of variability. For intracellular staining, cells were first incubated with LIVE/DEAD fixable violet dead cell stain (Invitrogen) for 30 min on ice, then fixed with fixation/permeabilization buffer (eBioscience) for 20 min on ice. The amount of IgG and goat anti-human IgG used were the same as described for the cell surface staining. Washing and fixation were performed using reagents and a protocol provided by the manufacturer. Cells were analyzed by flow cytometry within 48 hours. Cell populations were analyzed using an LSRII flow cytometer (BD Biosciences). Forward and side scatter gating was used to exclude cell fragments and debris. Dead cells were eliminated from the analysis through the use of the viability reagent. Binding of IBM-derived IgG to the cell lines (median fluorescence intensity) was compared to cells stained with healthy donor and control-derived IgG. Populations were compared using the two-tailed Mann-Whitney U test.

### Immunohistochemistry

Frozen tissue sections (20 µm) of mouse quadriceps were provided on microscope slides. Prior to immunohistochemistry, the tissue was fixed in acetone at −20°C for three minutes and air dried at room temperature. Sections were rinsed in PBS and blocked with PBS containing 5% goat serum for one hour. After incubation with 50 µg/ml of IgG for four hours at room temperature, slides were rinsed three times in PBS followed by incubation with goat anti-human IgG conjugated to AlexaFluor 488 (1∶1000; Molecular Probes) for 1 hour. Slides were rinsed, stained with the nuclear dye DAPI (1 µg/ml; Sigma) for 10 minutes, rinsed and mounted with FluorSave™ Reagent (Calbiochem). Fluorescent images were captured with a Zeiss AxioScope.

### Expression of Recombinant IgG

Recombinant IgG was prepared from single plasma cells isolated from IBM muscle tissue specimens and controls using an approach similar to that, which has been described previously, although minor modifications were introduced. Individual cells were isolated from tissue sections using laser capture micro-dissection [12], [21]. Single cell PCR was used to amplify the immunoglobulin variable heavy and light chains [21], [22]. These products were subsequently directionally sub-cloned behind the CMV promoter into a pcDNA3.3-based vector constructed in-house to harbor the human immunoglobulin constant domains (heavy and light chains). The heavy chain vector was modified to contain a C-terminal affinity tag (HA-hemagglutinin). The final constructs were engineered such that the rIgG produced did not include any non-native residues that can arise from inclusion of restriction enzyme sites and other engineering artifacts. Expression was achieved by transfecting a heavy and light chain-containing plasmid pair into CHO-S cells that have been adapted to grow in suspension and in a media that does not contain protein or require the addition of serum. Following transfection, cells were propagated for 6 days, and then the supernatant (containing the secreted rIgG) was collected and applied to a protein-G Sepharose column, which yielded purified rIgG upon elution [23], [24]. The rIgG-6, 6Hd1, 6Hd2 and 6HA1 were all obtained from the same muscle specimen, rIgG-5, rIgG-2 and rIgG-86H was each derived from unique muscle specimens. Serum samples matching these biopsy specimens were not available. Six rIgGs were used for the flow cytometry analyses (rIgG-2 was not included). Two (rIgG-5 and rIgG-2) were used for the immunoblotting.

### Immunoblotting and Mass Spectrometry

Human muscle tissue lysates (25 µg) were separated on 12% SDS-PAGE gels. Proteins were transferred to nitrocellulose membranes, then blocked in TBS-T (Tris buffered saline pH7.4, 0.05%Tween) containing 5% non-fat dry milk for 1 hour. The membranes were then incubated with recombinant IgG (10 µg/ml) overnight at 4°C. The membranes were washed 3 times with TBS-T and incubated with a HRP-conjugated anti-HA antibody (Roche) diluted 1∶500 in blocking buffer for one hour, washed as above then binding detected using enhanced chemiluminescence (ECL) Western blotting reagents (GE Healthcare). Bands were visualized on autoradiography film.

For mass spectrometry, human muscle tissue lysates were separated by SDS-PAGE, and then the protein bands were visualized with Coomassie staining. Bands of interest were excised from the gel, reduced, alkylated and digested with trypsin. The resulting peptides were extracted with organic solvents and concentrated to a fixed volume. The peptides were then applied to a capillary reverse phase (Phenomenex, Inc. Jupiter C_18_) high performance liquid chromatography (HPLC) column. Individual peptides were eluted with a linear gradient from 0–40% acetonitrile in formic acid (0.1%) using a Waters NanoAcuity HPLC and autosampler. The HPLC column was physically coupled to the tandem mass spectrometer in a nanospray configuration, such that the column eluate flowed directly into an ion trap mass spectrometer (ThermoFisher LTQ). For protein identification, ThermoScientific SEQUEST software was used to identify peptides. We narrowed our list of candidate proteins by selecting proteins of 50–60 kDa (near the size of the band in the immunoblot).

### ELISA

A 96-well plate was coated (250 ng/well) with commercially acquired Desmin and Vimentin (GenWay Biotech, San Diego, CA). The wells were blocked with PBS +2% BSA followed by addition of recombinant IgG at 10 µg/ml. Serial three-fold dilutions of the recombinant IgG were made to obtain a binding curve. Bound immunoglobulin was detected with a monoclonal peroxidase conjugated anti-HA antibody. The HRP-labeled secondary antibody was visualized using the peroxidase substrate, TMB (100 µl/well). The addition of 100 µl of 1 M hydrochloric acid stopped color development. Absorbance was measured at 450 nm on a µQuant™ Microplate Spectrophotometer.

### Statistical Analysis

Median fluorescence intensity (MFI) of different groups was compared using the Mann-Whitney test. All statistical analysis was performed using Prism 4.02 software (Graph Pad).

## Results

### IBM Serum-derived IgG Binds Muscle-associated Self-antigens

**Figure 1:**
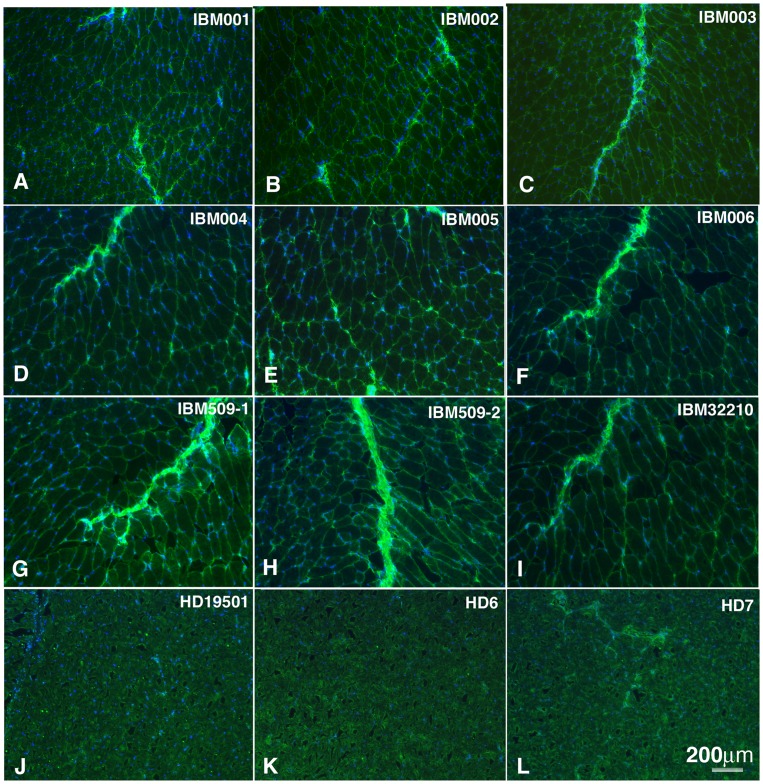
Immunostaining of muscle tissue with serum-derived IgG. Immunostaining of mouse muscle tissue demonstrates IBM-derived IgG positively label muscle tissue sections (A–I). The identity of the IgG specimens is shown in the top right corner of each panel. Staining is present within the endomysial space (green). Nuclei are stained in blue. In sections (A, C, D, F, G, H and I) IBM-derived IgG labeling can be seen in the endomysial space. In six of these samples binding was also observed in the perimysium (C, D and F–I). Tissue, from adjacent sections, stained with IgG derived from healthy donor (HD) serum (J–L) is free of any labeling.

To assess whether IBM serum-derived antibodies recognize self-antigens, we purified IgG from the serum of IBM patients and control subjects and looked for binding to muscle tissue sections by immunohistochemistry. To evaluate IgG binding to muscle antigens by immunohistochemistry, murine-derived tissue was favored over human as the latter provides high background due to endogenous immunoglobulin, which would be detected with our anti-human Ig secondary detection reagent. Moreover, purified immunoglobulin was used in preference to serum as the latter often produces a signal that includes nonspecific binding and does not allow precise control over the concentration of IgG among different specimens used in the assay. All nine IBM serum-derived IgG specimens demonstrated binding to muscle tissue sections with strong staining to the endomysial membrane (Figure 1A–I). In six of these samples binding was also observed in the perimysium (Figure 1C, D and F–I). In contrast, no binding was observed to adjacent tissue sections that were stained with serum-derived IgG from healthy donors (Figure 1J–L). Binding by IBM and control IgG was not observed when brain tissue sections were used (not shown). These data suggest that the peripheral circulation of IBM patients includes autoantibodies directed towards self-antigens that are present in muscle tissue.

### IBM Serum-derived IgG Binds to the Muscle Cell Lines CCL-136 and CRL-1598

**Figure 2:**
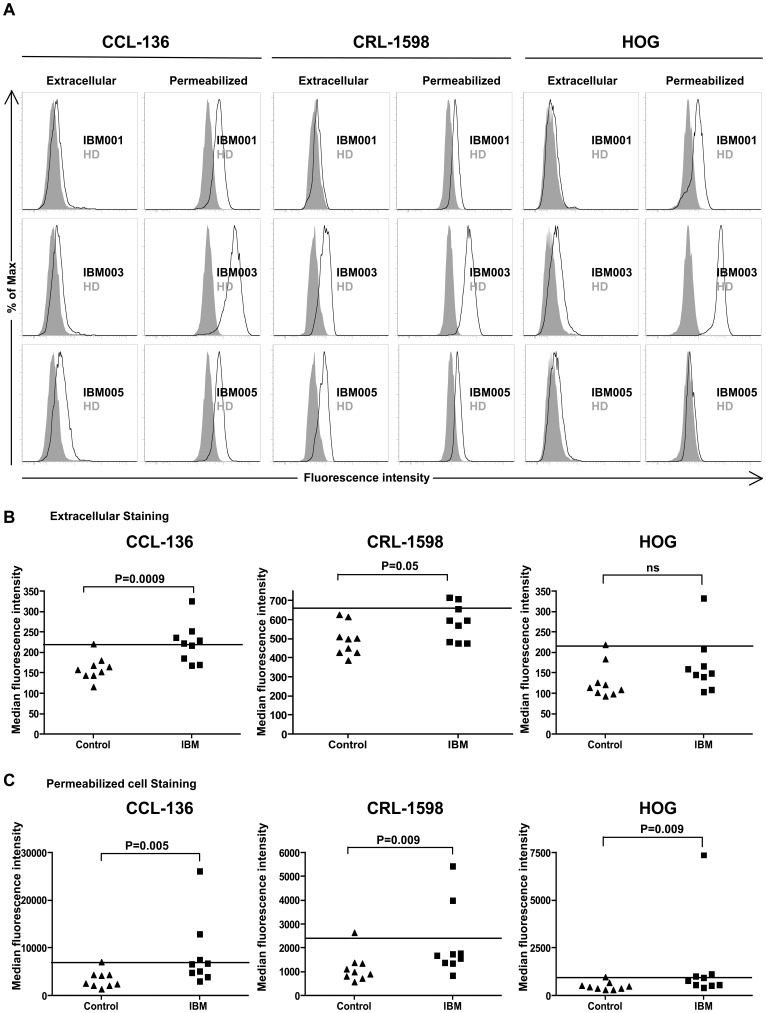
Cell-based assays to evaluate serum autoreactivity. A flow cytometry-based assay was used to evaluate IBM serum-derived IgG binding to human muscle-derived cell lines CCL-136, CRL-1598 and the human CNS-derived oligodendrocyte cell line, HOG. (A) Positive staining in the representative histograms is indicated by an increase in median fluorescence intensity (MFI) of an IBM-derived IgG (open curves) in comparison to a representative healthy control-derived IgG (closed curves). Specimen IBM001 binds to permeabilized muscle (CCL-136, CRL-1598) and non-muscle (HOG) cells but does not bind to cell surface components (extracellular staining). IBM003 binds to cell surface components of CRL-1598 while IBM005 binds to both CCL-136 and CRL-1598 cell lines. Both, IBM003 and IBM005 bind to permeabilized muscle cells (CCL-136 and CRL-1598). IBM003 binds to both intra- and extra-cellular components of the HOG line while IBM005 does not bind to either intracellular or extracellular components on the HOG cell line. The MFI values for all of the IBM serum-derived IgG specimens and controls are shown graphically. Assays performed with each of the three cell lines to measure binding to extracellular (B) and permeabilized cell lines (C) were collected for two different populations (healthy subjects and IBM patients). Each data point represents a single subject. The threshold line, above which specimens are considered positive, represents the mean +2 standard deviations of the normal healthy control cohort. A subset of IgG derived from IBM bind to self-antigens expressed within and on the surface of the cell lines tested.

To complement the IBM serum autoantibody binding observed with the muscle tissue sections, we measured serum IgG binding to cell surface determinants on human muscle-derived cell lines by flow cytometry (Figure 2A). The human rhabdomyosarcoma cell lines CCL-136 and CRL-1598 and a human oligodendrocyte-derived cell line (HOG) were used to test binding to self-antigens expressed by muscle (former two) and non-muscle cells (latter) respectively (Figure 2A). A subset (5 of 9 (56%)) of the IBM serum-derived IgG demonstrated binding to the cell surface of the muscle cell lines CCL-136, while 2 of 9 (22%) bound to the CRL-1598 cell line (Figure 2B). Healthy donor derived IgG (n = 9) showed no effective binding to either muscle cell line (Figure 2B). The binding of the IBM serum-derived IgG to both the cell lines CCL-136 (p = 0.0009) and CRL-1598 (p = 0.05) was significantly different from that of the healthy donor cohort (Figure 2B).

One IBM-derived IgG specimen (1 of 9 (11%)) bound to the surface of human brain-derived HOG cell line (Figure 2B). This specimen was among those that also bound to the CCL-136 and CRL-1598 cell line, suggesting that these cell lines may share an antigen recognized by this IBM serum specimen or that the serum harbors multiple populations of autoantibodies of different specificities. Healthy donor derived IgG (n = 9) showed no effective binding to the surface of the HOG cell line (Figure 2B). In addition to cell surface staining, staining was also performed on the same cell lines following permeabilization to allow for binding to intracellular antigens. A subset of IBM serum IgG demonstrated binding (Figure. 2C) to permeablized CCL-136 (3 of 9 (33%)) and CRL-1598 (2 of 9 (22%)) cell lines. The binding of the IBM serum-derived IgG to the cell lines CCL-136 (p = 0.005), CRL-1598 (p = 0.009) and HOG (p = 0.009) was significantly different to that of the healthy donor cohort. These IBM specimens were among the six that also recognized antigens expressed on the surface of CCL-136 and CRL-1598 cells. Healthy donor derived IgG (n = 9) showed minimal binding to both permeabilized muscle cell lines and the HOG cell line (Figure 2C).

Taken together, these data further suggest that the peripheral circulation of patients with IBM, analogous to other autoimmune conditions, includes autoantibodies that recognize both extra- and intracellular self-antigens expressed by human cells, including, but not limited to, those derived from muscle.

### Plasma Cells that Populate IBM Muscle Tissue Produce Antibodies That Recognize Self-antigens

**Figure 3:**
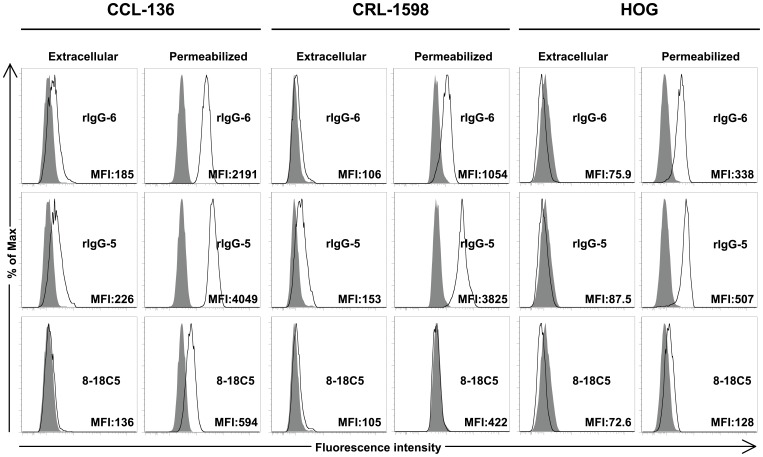
Cell-based assays to evaluate muscle-derived recombinant IgG autoreactivity. Recombinant IgG was prepared from single muscle tissue-associated plasma cells. A flow cytometry-based assay was then used to evaluate the binding of this rIgG to human muscle cell lines CCL-136, CRL-1598 and the human CNS-derived oligodendrocyte cell line, HOG. Representative positive staining shown in the histograms is indicated by an increase in median fluorescence intensity (MFI) of the IBM-derived rIgG (open curves) in comparison to staining with the secondary antibody alone (anti-human IgG-Alexa Fluor 647). A humanized monoclonal antibody, 8-18C5, specific for myelin oligodendrocyte glycoprotein served as negative control. The evaluation of extracellular binding revealed a modest increase in MFI for rIgG-5 (open curves) and rIgG-6 (open curves) with respect to the secondary antibody for both muscle-derived cell lines, but not the CNS-derived cell line. Both rIgG-5 and rIgG-6 demonstrate detectable binding to all three permeabilized cell lines.

Having determined that IBM serum harbors self-reactive immunoglobulin, we next focused our study at the site of IBM tissue injury. Earlier studies, from our group and others, established that antigen-experienced plasma cells reside in IBM muscle tissue [7], [11], [12]. Thus, we reasoned that autoantigens present in the damaged tissue might be the target of the antibodies produced at this site. To explore this possibility we isolated single plasma cells, directly from IBM-derived muscle tissue sections using laser capture microdissection and from these cells, prepared a series of recombinant immunoglobulins. Thus, each rIgG represents the IgG produced by a single plasma cell residing in the muscle tissue. These rIgG were used to assess whether IBM tissue-associated plasma cells produce antibodies that recognize self-antigens. Flow cytometry of the muscle-derived cell lines CCL-136 and CRL-1598 was used to initially evaluate rIgG binding. A humanized version of the monoclonal antibody 8-18C5 [23], a widely used anti-myelin oligodendrocyte glycoprotein (MOG) monoclonal antibody was used as a negative control as MOG is not expressed by these cells (Figure 3). Of the six IBM-derived recombinant IgGs examined, two rIgGs (rIgG-5 and rIgG-6, obtained from two different IBM patients) demonstrated modest binding to cell surface antigen(s) on CCL-136 and CRL-1598 relative to the control rIgG (Figure 3). The human oligodendrocyte-derived cell line (HOG) was used as a non-muscle control. Both rIgG-6 and rIgG-5 did not bind to cell surface antigen(s) on the HOG cell line (Figure 3). Curiously, when staining was performed on these same cell lines following permeabilization, strong binding was observed with the two rIgGs (rIgG-5 and rIgG-6) that showed modest binding to extracellular determinants (Figure 3). This binding was not restricted to muscle-derived cell lines as both antibodies also bound to the permeabilized HOG cell line suggesting that these antibodies may bind to an antigen shared by all three cell lines. Binding of control rIgG 8-18C5 to permeabilized cell lines was considerably lower than that observed with IBM-derived rIgG-5 and rIgG-6. These data suggest that the antigen-experienced plasma cells, associated with injured IBM muscle tissue produce antibody that can recognize self-antigens.

### Plasma Cells at the Site of IBM Tissue Injury Recognize a Muscle-associated Antigen

**Figure 4:**
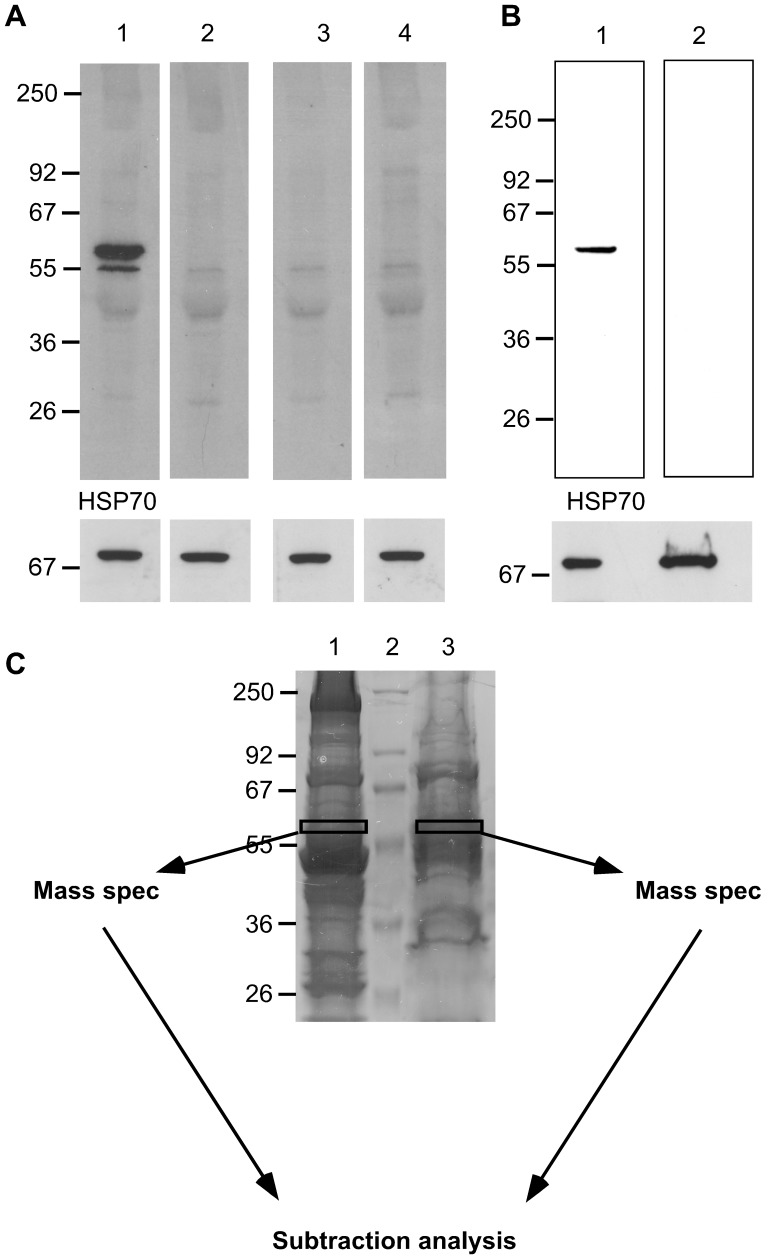
Autoantigen isolation and identification strategy. Recombinant IgG derived from an IBM muscle plasma cell was used to isolate an unidentified target antigen. Muscle tissue homogenates (25 µg) were resolved by SDS-PAGE, then stained with coomassie brilliant blue or transferred to nitrocellulose. Molecular weight markers (kDa) are indicated on the left of all panels. An immunoblot (A) of muscle tissue homogenate probed with rIgG derived from a single IBM-tissue associated plasma cell (rIgG-5) demonstrated binding to a candidate antigen (lane 1). Binding was not present with the rIgG-2 (lane 2), derived from a different muscle specimen or with control antibodies, D3a (lane 3) and 18/2 (lane 4) that were derived from a healthy donor and from a patient with lupus respectively. A second immunoblot (bottom) probed with anti-HSP70 served as a loading control. (B) Two separate human skeletal muscle homogenates prepared using either stringent (lane 1) or mild protein extraction conditions (lane 2) were resolved by SDS-PAGE gel, and then transferred to nitrocellulose. Immunoblotting with rIgG-5 (lane 1), demonstrated the target antigen was present when the homogenate was prepared with the stringent extraction, but not with the mild conditions (lane 2). An immunoblot (bottom) with HSP70 served as a loading control. (C) To isolate the candidate antigen, the two homogenates were separated by SDS-PAGE**,** then bands with the approximate molecular weight of the candidate antigen were excised from the gel. Lane 1, muscle homogenized with the stringent extraction procedure; Lane 2, molecular weight markers; Lane 3, muscle homogenized with the mild extraction procedure. These excised proteins were digested with trypsin and the resulting peptides separated by reverse-phase chromatography, then applied to a mass spectrometer. Proteins present in lane 3 were subtracted from those identified in lane 1 to reduce the complexity of the mass spectrometry data set.

Our observation that several of the rIgG derived from IBM muscle specific plasma cells recognized self-antigens, prompted us to search for their antigenic targets. We chose to focus on rIgG-5 because it provided the strongest binding to permeabilized cells, suggesting that the target of rIgG-5 may be an intracellular antigen. We used a human muscle tissue lysate as an antigen source, as opposed to a muscle-derived cell line, as the latter would include a more extensive representation of cellular components. Immunoblots of tissue lysate with IBM-derived rIgG-5 revealed a doublet near 55 kDa (Figure 4A, lane 1) that was not present in the blots probed with a second rIgG derived from a plasma cell (rIgG-2, lane 2) harbored by a different muscle specimen (Figure 4A) or with two control rIgG (lanes 3 and 4). This reactivity appeared to be restricted to muscle tissue as immunoblots performed with rIgG-5 on brain and spleen tissue lysates failed to identify any bands (not shown).

To identify the target of rIgG-5 we took advantage of the harsh extraction conditions required to solubilize the unknown target antigen. As shown in Figure 4B, immunoblot analysis using rIgG-5 on a muscle tissue homogenate prepared using harsh extraction conditions (lane 1) identified our band of interest that was not present in a muscle lysate prepared using milder conditions (lane 2). To identify this protein target, both lysates were separated by SDS-PAGE (Figure 4C) and bands corresponding to the size of our protein of interest were extracted and analyzed by mass spectrometry. By subtracting proteins identified using the mild extraction conditions (in which the target was not present) from those identified using the harsher conditions, we narrowed our list of candidates to eight proteins. These included Desmin, Myotilin, Tubulin, and Vimentin. We chose to focus on Desmin because a total of 29 different peptides were identified, each of which matched those found in human Desmin. By contrast Vimentin had six, Myotillin five and Tubulin only two.

**Figure 5:**
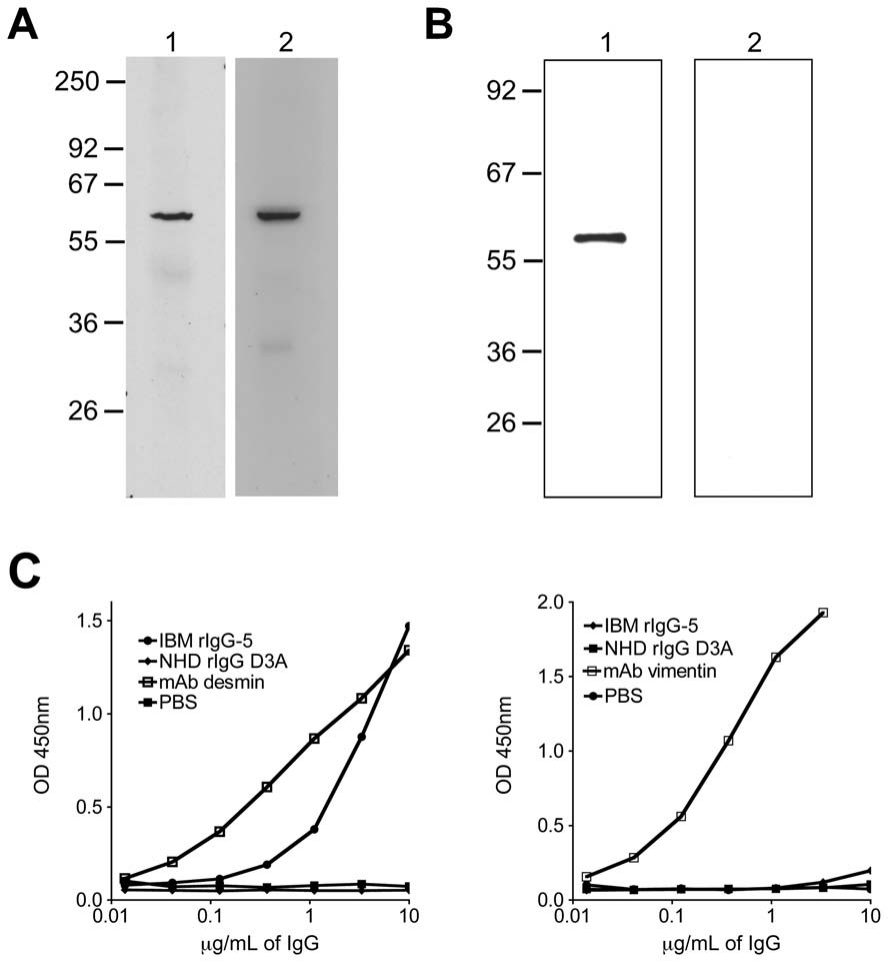
Immunoassays evaluating recombinant IgG binding to Desmin. Independent immunoassays were used to demonstrate that recombinant immunoglobulin derived from an IBM muscle-associated plasma cell (rIgG-5) binds to human Desmin. (A) Human muscle homogenates were resolved by SDS-PAGE then transferred to membranes that were probed with IBM-derived recombinant rIgG-5 (lane 1) or a commercial anti-Desmin monoclonal antibody (lane 2). (B) Purified human Desmin was resolved by SDS-PAGE then transferred to membranes that were probed with IBM-derived rIgG-5 (lane 1) or rIgG-D3a (lane 2), which was derived from an immature antigen-inexperienced peripheral human B cell. (C) ELISA assays demonstrating IBM-derived immunoglobulin (rIgG-5) binding to the human muscle protein Desmin. ELISA plates were coated with either Desmin (left panel) or Vimentin (right panel) then probed with IBM rIgG-5 and a rIgG (D3a) derived from a single B cell from a normal healthy donor. Monoclonal antibodies (mAb) specific for each antigen were used as positive controls.

To assess whether rIgG-5 binds Desmin, we probed immunoblots of human muscle homogenate with both the IBM-derived rIgG-5 recombinant immunoglobulin and a commercial anti-human Desmin monoclonal antibody. Both antibodies produced a single band of identical size (Figure 5A) that corresponded to the molecular weight of human Desmin. Next, we probed immunoblots of purified human Desmin with rIgG-5 and a control rIgG derived from a normal immature B cell (rIgG-D3a). The rIgG-5 recognized Desmin (lane 1) in the immunoblot while the control rIgG (lane 2) did not (Figure 5B). We further established Desmin as the target of rIgG-5 using ELISA with purified Desmin and Vimentin. We chose Vimentin as a control due to its close homology (69% at the amino acid level) with Desmin. Commercially available monoclonal antibodies to Desmin (Figure 5C left panel) and Vimentin (Figure 5C right panel) bound to their respective antigens over IgG concentrations ranging from 0.02–10 µg/mL. Control-derived rIgG-D3a did not bind Desmin or Vimentin. IBM-derived rIgG-5 bound Desmin over a range of antibody concentrations from 0.2–10 µg/mL, but did not bind to Vimentin (Figure 5C). These data demonstrate that the IBM-derived clone rIgG-5 recognizes human Desmin and indicates that plasma cells at the site of IBM tissue injury can produce autoantibodies.

## Discussion

The current study was initiated to investigate the humoral immune response in IBM and determine whether a component of it is directed towards self-antigens. In this work we have provided support for the presence of a genuine humoral autoimmune response both in the periphery and at the site of tissue damage in IBM patients. The historical view of this disease has been that the pathology is mediated by skeletal muscle degeneration and, in part, by CD8+ T cells that are known to populate the damaged tissue [1], [9], [25]–[28]. The data presented here contributes to an emerging body of evidence [7], [12], [15] that suggests humoral autoimmunity also occurs in patients with IBM. We have shown that immunoglobulins that recognize self-antigen(s) can be detected in the peripheral circulation of patients with IBM. Two different, complementary techniques (immunohistochemistry and flow cytometry) were used to demonstrate that such autoantibodies exist. We extended our analysis to investigate the specificity of the immunoglobulin expressed by plasma cells that populate the damaged muscle tissue of patients with IBM. That we identified a self-antigen as the target of the immunoglobulin produced by IBM muscle tissue-associated plasma cells lends further evidence to the concept that IBM includes humoral autoimmunity. It further indicates that this autoimmune response is active, at least in part, within the environment associated with tissue injury. It should be noted that the co-existence of peripheral and localized immune responses have been demonstrated in a number of autoimmune diseases that include a humoral component. Autoantibodies have been detected in the periphery and at the site of tissue injury in rheumatoid arthritis where autoantibodies are observed in both the circulation and the synovia of the joints [29] and in neuromyelitis optica (NMO) where autoantibodies are observed both in the periphery and in the central nervous system [30].

While this study demonstrates that part of the humoral immune response in IBM patients is directed towards self-antigens, we must however stress that it remains unknown whether this response actively contributes to disease pathogenesis or simply represents an epiphenomenon. Furthermore, it remains likely that IBM is a disease with multiple antibody specificities as seen with the other inflammatory myopathies dermatomyositis and polymyositis, where a wide spectrum of antigens [31], including nuclear and cytoplasmic components have been identified. The heterogeneous nature of the IBM humoral immune response is supported by previous studies reporting autoantibodies in IBM patients to a number of targets [31] with one study reporting that 44% of IBM patients display autoantibodies to one or more of nine non-disease specific targets [32]. However deciphering which autoantibodies are IBM specific will require some care as approximately 13% of IBM patients are reported to share coexisting conditions that may account for some of the observed reactivity [32], [33]. One recent study reported the identification of a humoral immune response in half of all IBM patients to a 43 kDa muscle protein [15] that appears to be disease specific.

In this study, we identified the intermediate filament, Desmin as the target of the immunoglobulin produced by a single plasma cell at the site of muscle tissue injury in one IBM patient. Interestingly, Desmin has been identified as an autoantigen in other autoimmune diseases [17]–[19] and is therefore not uniquely associated with IBM. However, relevant to the objectives of our study, the identification of this autoantibody-autoantigen pair provides evidence that self-reactive antibodies are produced by tissue infiltrating plasma cells. As Desmin is an intracellular protein it is unlikely that this reactivity represents an initiating event that leads directly to the development of IBM. Such intracellular autoantibodies are often useful as diagnostic or prognostic markers for disease. Yet, it is possible that this reactivity contributes to disease following initial muscle damage and the release of intracellular antigens. Interestingly, Desmin is upregulated in all three inflammatory myopathies [34]. However, it is unclear whether this upregulation is a degeneration-induced effect. That we found this reactivity in a single subject prohibits establishing a connection between Desmin and IBM. The presence of such autoantibodies may indeed not be a frequent event in this disease. Studies regarding the prevalence of autoantibodies to Desmin in patients with IBM, which will shed light on this, are underway.

### Conclusions

Taken together, our description of antibodies directed against self-antigens suggests that there is a humoral autoimmune component in IBM – at least in a subset of patients. Our results should trigger investigations that would set out to identify and validate additional autoantigens and determine whether such autoantibodies participate in disease pathology and if they can serve as useful diagnostic or prognostic biomarkers that are lacking for IBM. Additionally, investigations regarding the mechanism and functional effects of the humoral branch of the immune system in IBM pathophysiology need to be initiated.
